# X-box binding protein 1 induces the expression of the lytic cycle transactivator of Kaposi's sarcoma-associated herpesvirus but not Epstein–Barr virus in co-infected primary effusion lymphoma

**DOI:** 10.1099/vir.0.025494-0

**Published:** 2011-02

**Authors:** Imogen Yi-Chun Lai, Paul J. Farrell, Paul Kellam

**Affiliations:** 1 University College London, MRC Centre for Molecular Virology, Department of Infection, Division of Infection and Immunity, Windeyer Institute of Medical Science, 46 Cleveland Street, London W1T 4JF, UK; 2 Section of Virology, Imperial College Faculty of Medicine, St Mary's Campus, Norfolk Place, London W2 1PG, UK; 3 Wellcome Trust Sanger Institute, Wellcome Trust Genome Campus, Hinxton, Cambridge CB10 1SA, UK

## Abstract

Cells of primary effusion lymphoma (PEL), a B-cell non-Hodgkin's lymphoma, are latently infected by Kaposi's sarcoma-associated herpesvirus (KSHV), with about 80 % of PEL also co-infected with Epstein–Barr virus (EBV). Both viruses can be reactivated into their lytic replication cycle in PEL by chemical inducers. However, simultaneous activation of both lytic cascades leads to mutual lytic cycle co-repression. The plasma cell-differentiation factor X-box binding protein 1 (XBP-1) transactivates the KSHV immediate–early promoter leading to the production of the replication and transcription activator protein (RTA), and reactivation of KSHV from latency. XBP-1 has been reported to act similarly on the EBV immediate–early promoter Zp, leading to the production of the lytic-cycle transactivator protein BZLF1. Here we show that activated B-cell terminal-differentiation transcription factor X-box binding protein 1 (XBP-1s) does not induce EBV BZLF1 and BRLF1 expression in PEL and BL cell lines, despite inducing lytic reactivation of KSHV in PEL. We show that XBP-1s transactivates the KSHV RTA promoter but does not transactivate the EBV BZLF1 promoter in non-B-cells by using a luciferase assay. Co-expression of activated protein kinase D, which can phosphorylate and inactivate class II histone deacetylases (HDACs), does not rescue XBP-1 activity on Zp nor does it induce BZLF1 and BRLF1 expression in PEL. Finally, chemical inducers of KSHV and EBV lytic replication in PEL, including HDAC inhibitors, do not lead to XBP-1 activation. We conclude that XBP-1 specifically reactivates the KSHV lytic cycle in dually infected PELs.

## INTRODUCTION

The human gammaherpesviruses Epstein–Barr virus (EBV) and Kaposi's sarcoma-associated herpesvirus (KSHV) are associated with B-cell lymphomas and tumours of epithelial and endothelial origin, respectively. EBV and KSHV co-infection occurs in approximately 80 % of the non-Hodgkin's B-cell lymphoma, primary effusion lymphoma (PEL); the remainder being infected by KSHV alone. KSHV and EBV dually infected PEL have a subtly different pattern of B-cell gene expression compared with KSHV singly infected PEL (Fan *et al.*, 2005). In addition, the presence of EBV appears to potentiate the tumourigenicity of dually infected PELs in SCID mice (Trivedi *et al.*, 2004). How dually infected PEL arise is not understood, however, both KSHV and EBV latently infect PEL and normal B-cells.

EBV establishes a latent infection in memory B-cells *in vivo* (Babcock *et al.*, 1998, 1999; Souza *et al.*, 2005; Thorley-Lawson, 2001). A model for EBV infection suggests that, following saliva transmission, EBV infects IgD-positive antigen-naïve B-cells in the nasopharyngeal lymphoid tissue (Joseph *et al.*, 2000) and induces B-cell proliferation through the expression of virus latent growth programme genes (Thorley-Lawson & Gross, 2004). The EBV-positive lymphoblasts subsequently express the default/latency II gene-expression programme (Thorley-Lawson, 2001; Thorley-Lawson & Gross, 2004), finally entering the memory B-cell repertoire, where the virus silences its gene expression allowing lifelong persistence (Gires *et al.*, 1997; Panagopoulos *et al.*, 2004). The type of B-cell and the mechanism of latent colonization by KSHV are not known.

Horizontal virus transmission requires either EBV and/or KSHV to reactivate from latency with infectious EBV and KSHV being transmitted by saliva. The molecular events that lead to virus reactivation have been studied extensively. For EBV the reactivation of the lytic cycle is mediated by two viral proteins, BZLF1 (ZTA) and BRLF1 (RTA) (Amon *et al.*, 2004; Chang & Liu, 2000; Yuan *et al.*, 2006). BZLF1 is the key immediate–early protein in EBV (Bryant & Farrell, 2002; Countryman & Miller, 1985), with B-cell receptor (BCR) cross-linking able to activate the BZLF1 promoter (Zp) but not the BRLF1 promoter (Rp). Induction of Zp is therefore the first event of EBV lytic reactivation (Amon *et al.*, 2004), and BZLF1 expression alone is sufficient to initiate the entire EBV lytic cycle (Countryman & Miller, 1985; Rooney *et al.*, 1989). For KSHV, the virus immediate–early replication and transcription activator protein (RTA) is both necessary and sufficient to activate the virus lytic cycle. RTA is able to transactivate its own promoter and those of many viral genes, leading to the KSHV lytic gene expression cascade (Lukac *et al.*, 1998).

The authentic cellular mediators that lead to the induction of EBV BZLF1 (ZTA) and KSHV RTA (K-RTA) are less clear. Previously, we and others showed that the activated B-cell terminal-differentiation transcription factor X-box binding protein 1 (XBP-1s) is sufficient to initiate the lytic cycle of KSHV in PEL cell lines (Dalton-Griffin *et al.*, 2009; Sun & Thorley-Lawson, 2007; Wilson *et al.*, 2007). XBP-1s binds to and transactivates the promoter of KSHV RTA. XBP-1s expression is essential for terminal differentiation of plasma cells (Reimold *et al.*, 2001). XBP-1 retains a 26 nt intron when expressed (XBP-1u, unspliced) that results in a frame shift preventing the translation of an active transcription factor. Under endoplasmic reticulum (ER) stress or B-cell terminal differentiation, the 26 nt intron of XBP-1u mRNA is removed to allow the translation of the active form, XBP-1s (XBP-1 spliced) (Calfon *et al.*, 2002). EBV lytic replication is induced in plasma cells (Laichalk & Thorley-Lawson, 2005); however, EBV infection of multiple myeloma cell lines does not result in lytic EBV replication even though XBP-1s is abundantly expressed in these cells (Anastasiadou *et al.*, 2009). Interestingly, other studies have also suggested that XBP-1s is able to bind to and transactivate the EBV BZLF1 promoter (Bhende *et al.*, 2007; Sun & Thorley-Lawson, 2007). Although XBP-1 can be transiently activated following BCR cross-linking (McDonald *et al.*, 2010), XBP-1s alone may not be sufficient to induce expression of EBV ZTA as protein kinase D (PKD) may also be required (Bhende *et al.*, 2007).

In dually infected PEL a selective lytic switch has been proposed that leads to the induction of one virus over the other (Miller *et al.*, 1997). Indeed K-RTA can only induce the KSHV lytic cycle and BZLF1 can only induce the EBV lytic cycle in PEL. The selective switch can be maintained by the induced virus blocking the lytic replication of the other latent virus (Xu *et al.*, 2007). If both K-RTA and BZLF1 are expressed at the same time, physical interactions promote mutual inhibition of the two transcription factors (Jiang *et al.*, 2008). Here we investigate the mechanism that underlies the initial induction of K-RTA and BZLF1 in dually infected PEL (Miller *et al.*, 1997). We show that XBP-1s alone induces the KSHV lytic cycle but does not induce either EBV BZLF1 or BRLF1 in PEL cell lines; it also does not induce either of the EBV immediately–early proteins in EBV-positive Burkitt's lymphoma cell lines (BLs). Additionally, we show that XBP-1s does not activate the BZLF1 promoter of EBV in HEK 293T and HeLa cells. Overexpressing XBP-1s in both PEL and BLs does not increase the level of BZLF1 and BRLF1 mRNA. These results suggest that XBP-1s activates the KSHV lytic cycle alone in EBV co-infected PEL cells.

## RESULTS

### XBP-1s overexpression in PEL cell lines results in KSHV RTA expression but not EBV BZLF1 expression

**Fig. 1.**
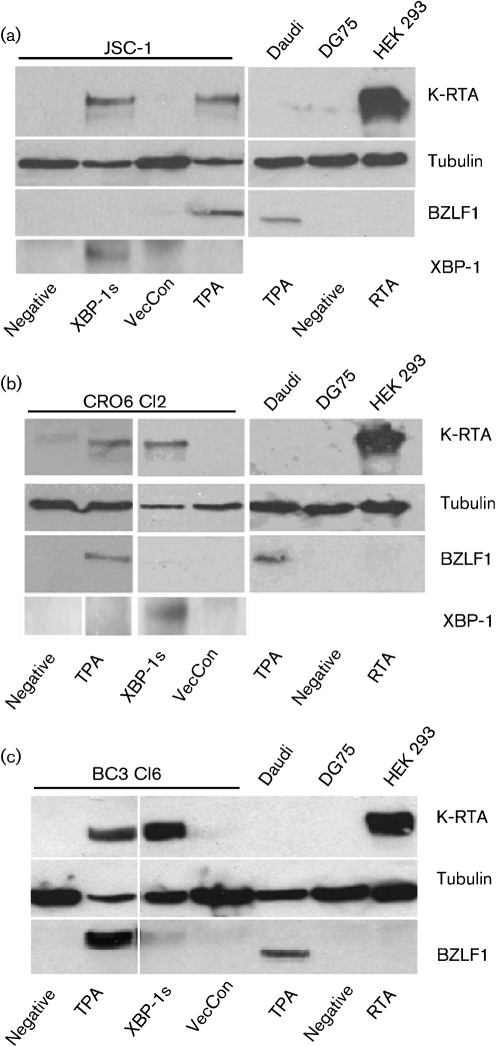
XBP-1s does not activate EBV lytic gene expression in PEL cell lines. (a) JSC-1, a KSHV and EBV double-positive PEL cell line, expressed K-RTA and BZLF1 after treatment with TPA, but only K-RTA was expressed following XBP-1s transduction. (b) BC3 cl6, and (c) CRO6 cl2 are EBV-superinfected KSHV-positive PEL cell lines and showed a similar pattern with XBP-1s transduction only resulting in K-RTA expression. TPA was able to induce both K-RTA and BZLF1. The Daudi cell line treated with TPA and K-RTA-transfected HEK 293T cells acted as positive controls for the BZLF1 and K-RTA antibody staining, respectively. XBP-1s expression was detected using an XBP-1s specific antibody. VecCon, vector control.

In order to examine the ability of XBP-1s to induce the EBV lytic cycle, we used lentiviral vectors for the transient delivery of XBP-1s into different PEL cell lines. The effect of XBP-1s overexpression on both KSHV and EBV were evaluated by Western blotting against the KSHV immediate–early gene K-RTA and the EBV immediate–early gene BZLF1 (ZTA), respectively. Overexpressing XBP-1s in JSC-1 cells, a KSHV and EBV co-infected PEL, induced K-RTA expression. However, no EBV BZLF1 protein expression was detected, despite the ability of *O*-tetradecanoyl-phorbol 13-acetate (TPA) to induce the expression of K-RTA and BZLF1 in JSC-1 simultaneously (Fig. 1a).

It is possible that EBV in PEL cell lines is in some way defective or non-responsive to XBP-1s. Therefore, we repeated the experiment with BC3 clone 6 (BC3 cl6) and CRO6 clone 2 (CRO6 cl2); two cell lines that were produced by superinfecting KSHV single-positive PEL cell lines with replication-competent EBV–GFP virus (Xu *et al.*, 2007). Western blot analysis showed identical results with the induction of K-RTA but no expression of BZLF1 (Fig. 1b, c) after overexpressing XBP-1s in these cells. TPA treatment again resulted in induction of K-RTA and BZLF1. In all cases, 60–80 % of PEL cells were transduced by the XBP-1s-expressing lentivirus (data not shown). Expression of XBP-1s was confirmed by using Western blot analysis (Fig. 1a, b). Therefore, XBP-1s induces K-RTA expression in PELs, but does not induce BZLF1 expression.

### Overexpression of XBP-1s in BL cells does not induce EBV BZLF1 expression

**Fig. 2.**
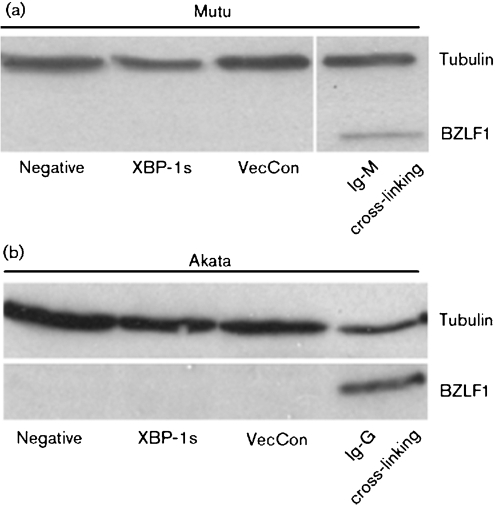
Burkitt's lymphoma (BL) cell lines do not express BZLF1 after transduction with XBP-1s. (a) Mutu and (b) Akata, both EBV-positive BL cell lines, were transduced with XBP-1s and vector control (VecCon) but BZLF1 expression was not detected. B-cell surface cross-linking (BCR) with human Ig is used as positive control to induce BZLF1 expression: (a) Mutu, with Ig-M. (b) Akata, with Ig-G.

It is possible that the presence of KSHV in PEL affects the ability of XBP-1s to induce BZLF1 expression. We therefore expressed XBP-1s in BLs, which are only EBV positive. Using BCR cross-linking we could induce BZLF1 expression (Fig. 2). However, overexpressing XBP-1s in two different BL lines, Mutu and Akata, did not induce BZLF1 expression despite good transduction efficiencies of 10–25 %. This suggests that XBP-1s does not transactivate the BZLF1 promoter in B-cell lymphomas regardless of the presence of KSHV.

### Overexpression of XBP-1s in BL or PEL cells does not induce EBV BRLF1 and BMRF1 expression

**Fig. 3.**
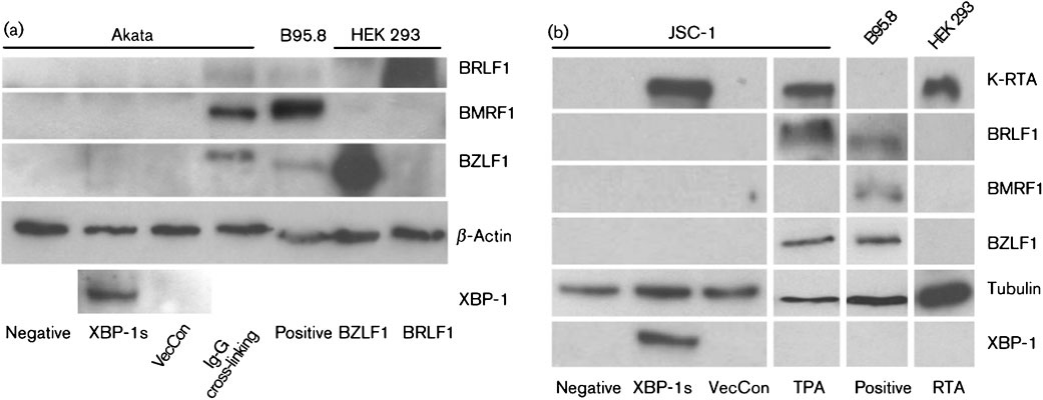
Expression of XBP-1s does not induce BRLF1 and BRLF1 protein expression in either BL or PEL cell lines. (a) Akata and (b) JSC-1 cell lines were transduced with XBP-1s and vector control with m.o.i. of 5 and 2, respectively (m.o.i. measured in JSC-1 cells). Neither BZLF1, BRLF1 nor BMRF1 expression were detected in either cell line. BCR cross-linking was used as a positive control for Akata to induce EBV lytic gene expression and TPA was used as a positive control for JSC-1 cell lines to induce KSHV and EBV lytic gene expression. Both positive controls showed strong BZLF1 expression, weaker BRLF1 expression, and, for Akata (a) only, expression of BMRF1 with BCR cross-linking. B95.8 cell line was used as a positive control for EBV immediate–early and early protein expression.

BRLF1 is a second EBV immediate–early protein whose expression is induced by BZLF1 during the EBV lytic cycle. Together these two proteins induce the rest of EBV lytic cascade. We examined the expression of BRLF1 after overexpressing XBP-1s in both BL (Akata) (Fig. 3a) and PEL (JSC-1) (Fig. 3b) cells. The expression of the EBV early protein BMRF1, also known as diffused early antigen (EA-D) (Bayliss & Wolf, 1981), was also examined by using Western blot analysis. BMRF1 transcription is activated by BZLF1 (Kenney *et al.*, 1992). However, in BL or PEL cell lines, overexpression of XBP-1s did not induce BZLF1, BRLF1 and BMRF1 expression. Interestingly, co-induction of K-RTA, BZLF1 and BRLF1 by TPA in PEL failed to induce BMRF1 expression, consistent with mutual co-repression of the full virus lytic cycle (Jiang *et al.*, 2008).

### EBV lytic-cycle promoters do not respond to XBP-1s

**Fig. 4.**
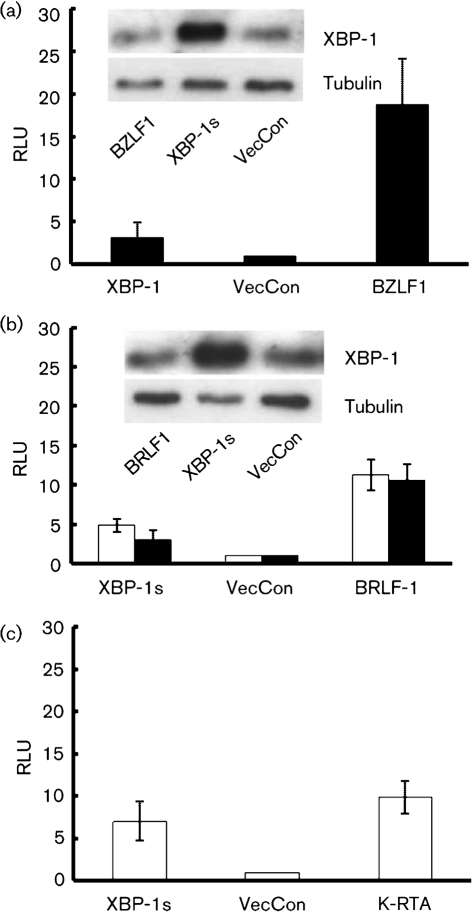
BZLF1 and BRLF1 promoter responses to XBP-1s. (a) XBP-1s weakly transactivates the Zp in a luciferase-reporter assay in HEK 293T cells. BZLF1 overexpression transactivates the Zp as a positive control. (b) BRLF1 and XBP-1s expression transactivate the Rp. The XBP-1s expression level in Zp- (d, insert) and Rp- (e, insert) transfected cells co-transfected with BZLF1, BRLF1 or XBP-1s was determined by Western blotting. (c) The K-RTA promoter was used as a positive control for XBP-1s expression and activity. The promoter is transactivated by both K-RTA and XBP-1s. RLU, Relative light units.

Previously, we showed that XBP-1s can transactivate the K-RTA promoter (Wilson *et al.*, 2007), thereby inducing the KSHV lytic cycle. We therefore determined whether the EBV BZLF1 or BRLF1 promoters can be transactivated by XBP-1s. A construct containing BZLF1 promoter fused to a luciferase reporter gene (McDonald *et al.*, 2010) was used to determine the response to XBP-1s in HEK 293T cells; however, we were unable to show strong XBP-1s activity on the BZLF1 promoter (Fig. 4a). The experiments were repeated using the BRLF1 promoter (Bhende *et al.*, 2007) in either the methylated or unmethylated form. Both forms of the BRLF1 promoter were transactivated by BRLF1 and had a lesser but detectable response to XBP-1s (Fig. 4b). The activity was therefore independent of the promoter methylation status. The activity of XBP-1s in this assay system was confirmed by using a KSHV RTA luciferase promoter reporter that responded to both KSHV RTA and XBP-1s (Fig. 4c). The expression of XBP-1s in the luciferase assay was confirmed by Western blot analysis (Fig. 4a, b insert).

### XBP-1 is unspliced in different B-cell lymphomas and splicing is not induced by TPA or  valporic acid (VPA)

**Fig. 5.**
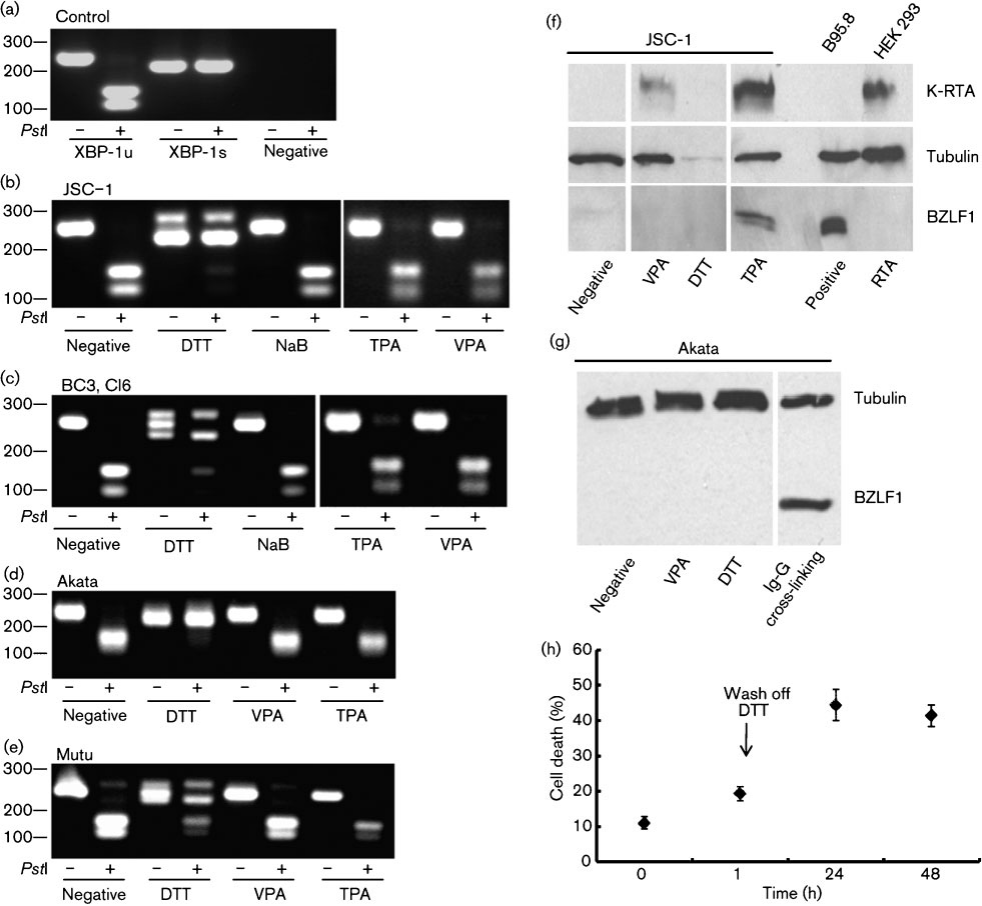
NaB, TPA and VPA do not induce XBP-1 splicing in PEL and BL cell lines. (a) XBP-1 is expressed as an unspliced (XBP-1u) form, but the spliced, active form (XBP-1s) can be detected following ER stress induction by the resistance to *Pst*I digestion of the XBP-1s PCR product. Treating the cells with DTT induces ER stress and XBP-1u splicing in all lymphoma cell lines (b–e). However, NaB, TPA and VPA treatment do not induce XBP-1 splicing in the lymphoma cell lines (b–e) even though VPA induces K-RTA expression in the PEL JSC-1 and TPA induces both K-RTA and BZLF1 in JSC-1 cells (f). VPA and DTT do not induce BZLF1 expression in the BL line Akata (g). DTT toxicity (h) results in PEL cell death and the absence of detectable proteins (f).

XBP-1u is expressed in both PEL and BL cell lines. DTT, a reducing agent able to induce the KSHV lytic cycle in KSHV-infected HEK 293T cells, can cause the splicing of inactive XBP-1u mRNA to active XBP-1s mRNA by promoting the unfolded-protein response (UPR) (Wilson *et al.*, 2007). As TPA can induce K-RTA and BZLF1 expression, and previous reports show other chemical agents can induce these viruses (Ye *et al.*, 2007), we determined whether they do so in part by inducing XBP-1splicing. DTT, TPA, VPA and sodium butyrate (NaB) (both histone deacetylase inhibitors) were used and XBP-1 splicing was assessed in the PEL cell line JSC-1 and BC3 cl6 cell line. Only DTT caused XBP-1 splicing in PEL cell lines despite TPA being capable of inducing KSHV K-RTA and EBV BZLF1 expression (Fig. 5b, c, f). We also examined the splicing status of XBP-1 in the BL cell lines Akata and Mutu. Again, XBP-1 is expressed as the unspliced inactive form and this only undergoes splicing in the presence of DTT (Fig. 5d, e).

### Induction of XBP-1 splicing by the UPR does not induce the KSHV and EBV lytic cycle

As DTT is capable of inducing XBP-1 splicing and the KSHV lytic cycle in HEK 293T cells (Wilson *et al.*, 2007), we examined the ability of DTT to induce the lytic reactivation of KSHV and EBV by Western blotting for the expression of K-RTA and BZLF1. Despite being able to cause XBP-1 splicing, DTT resulted in large amounts of cell death in PEL (Fig. 5h) After treating the cells with DTT for 10 min, JSC-1 cells lose membrane integrity and stain with trypan blue; after 48 h of incubation, more than 50 % of JSC-1 cells are dead (Fig. 5h). Therefore, and because of the toxicity of DTT in PEL cells, it is not possible to access the effect of XBP-1 splicing (Fig. 5f). The BL cell line Akata was more resilient to DTT treatment; however, despite the ability of DTT to induce XBP-1 splicing, BZLF1 was not expressed (Fig. 5g).

### Overexpression of XBP-1s or use of chemical agents does not induce expression of BZLF1 and BRLF1 mRNA in PEL or BL

Although XBP-1s overexpression did not induce detectable BZLF1 and BRLF1 protein expression in PEL or BL cell lines, the luciferase assays showed that XBP-1s weakly transactivated Zp and Rp in HEK 293T cells. We therefore performed a quantitative real-time PCR (Q-RT-PCR) to determine the mRNA levels of BZLF1 and BRLF1 in PEL and BL cell lines in response to XBP-1s and various chemical inducers of the KSHV and EBV lytic cycles.

**Fig. 6.**
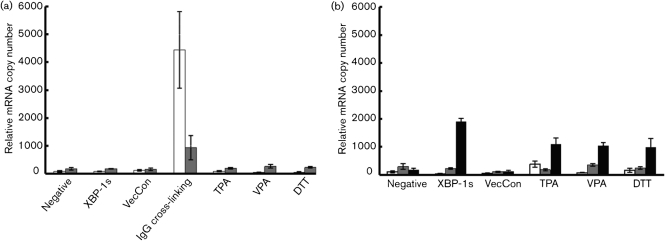
Overexpression of XBP-1s in PEL and BL cell lines does not increase the mRNA expression levels of BZLF1 and BRLF1. Q-RT-PCR was used to determine the expression of BZLF1 mRNA (open bars), BRLF1 mRNA (grey bars) and K-RTA mRNA (black bars) after transduction with XBP-1s lentivirus or treatment with chemical inducers for (a) Akata and (b) JSC-1 cell lines. (a) Neither XBP-1s overexpression nor TPA, VPA or DTT treatments were able to increase BZLF1 or BRLF1 mRNA expression. BCR cross-linking induced BZLF mRNA expression (*P*<0.05, two-tailed *t*-test). (b) In JSC-1 cells, expression of XBP-1s only increased K-RTA mRNA expression. TPA, VPA and DTT treatment also increased K-RTA mRNA expression significantly (*P*<0.05, two tailed *t*-test). TPA-treated JSC-1 cells expressed BZLF1 mRNA compared with negative control (*P*<0.05, two tailed *t*-test).

In Akata cells, BZLF1 and BRLF1 mRNA expression did not increase following XBP-1s expression or following TPA, VPA and DTT treatment (Fig. 6a). BCR cross-linking used as a positive control induced BZLF1 mRNA expression significantly (*P*<0.05, two-tailed *t*-test). The increase of BRLF1 mRNA is not statistically significant (*P*>0.05, two-tailed *t*-test) and is consistent with the lack of BRLF-1 protein expression (Fig. 3a). This is also consistent with previous work by Amon *et al.* (2004), showing that BCR-cross-linking transactivates Zp, but not Rp.

EBV BZLF1, EBV BRLF1 and KSHV K-RTA mRNA expression was determined in JSC-1 cells by using Q-RT-PCR (Fig. 6b). After XBP-1s transduction or VPA and DTT treatment, BZLF1 and BRLF1 mRNA expression was not significantly different from control cells (*P*>0.05, two-tailed *t*-test). However, the K-RTA mRNA level increased significantly in response to XBP-1s, TPA, VPA and DTT (*P*<0.05, two-tailed *t*-test). TPA induced a significant increase in the BZLF1 mRNA level, compared with the negative control (*P*<0.05, two-tailed *t*-test). These data confirm that XBP-1s alone does not transactivate the EBV Zp or Rp in PEL or BL cell lines.

### XBP-1s and protein kinase D together do not induce BZLF1 expression in PEL cell lines

**Fig. 7.**
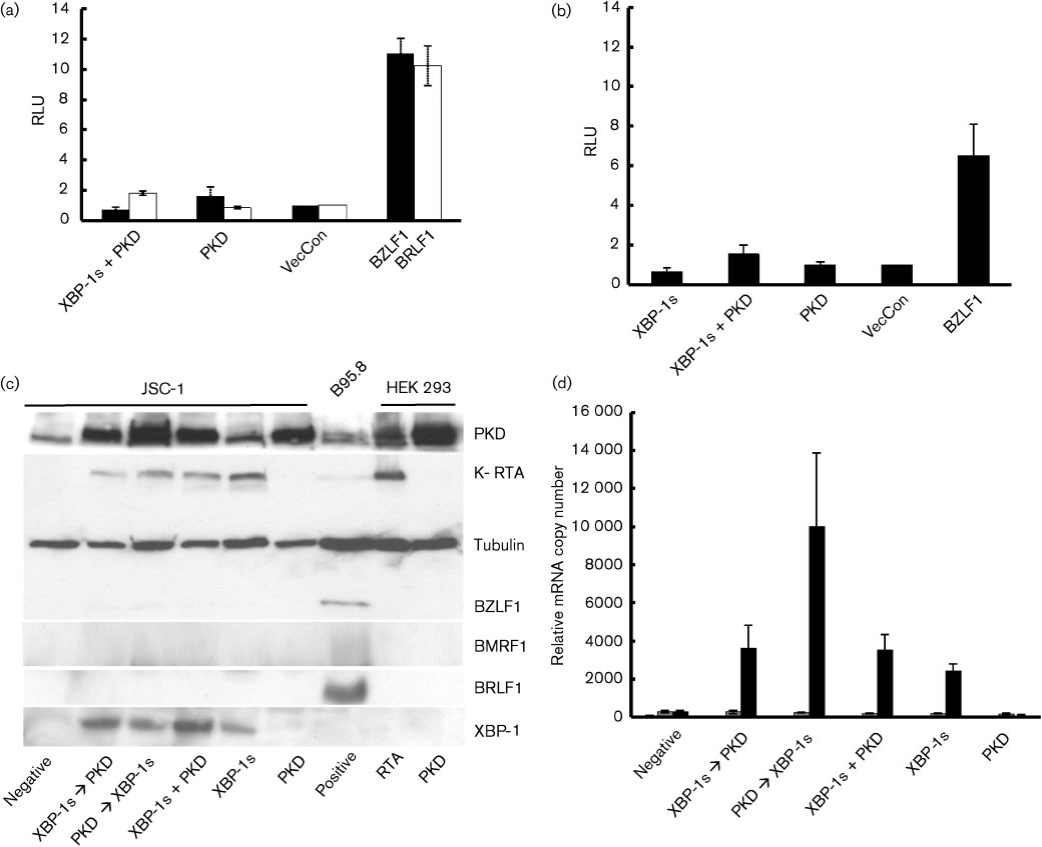
Co-expression of XBP-1s and active PKD does not transactivate Zp or induce BZLF1 expression. (a) HEK 293-T-cells were transduced with Zp (black bars) or Rp (open bars) luciferase-reporter vectors with either active PKD or XBP-1s together with active PKD. Active PKD alone shows weak Zp activity and together with XBP-1s weak Rp activity compared to control empty vector (VecCon) and BZLF1 or BRLF1 expression vectors. However, the increase of luciferase activity was not statistically significant compared to vector control (*P*>0.05, two-tailed *t*-test). (b) The Zp promoter assays were repeated in HeLa cells where only XBP-1s and PKD together showed weak promoter activity compared with BZLF1 overexpression. However, this was not statistically significant compared to vector control (*P*>0.05, two tailed *t*-test). RLU, Relative light units. (c) Transduction of JSC-1 cells with XBP-1s and active PKD either alone, in combination or sequentially (denoted by arrows) does not induce BZLF1, BRLF1 or BMRF1 expression in PEL, but all combinations that include XBP-1s induce K-RTA expression. (d) mRNA expression in JSC-1, measured by Q-RT-PCR after XBP-1s and/or active PKD transduction for BZLF1 mRNA (open bars), BRLF1 mRNA (grey bars) and K-RTA (black bars). Only K-RTA mRNA expression increased significantly (*P*<0.05, two tailed *t*-test) after XBP-1s transduction with or without PKD expression.

Previously Bhende *et al.* (2007) showed that XBP-1s alone was not sufficient to induce lytic reactivation of EBV and that PKD was also required. We therefore performed luciferase assays to investigate the effect of the combined expression of XBP-1s and a constitutively active PKD (pPKDm-IG). In HEK 293T cells, PKD alone weakly transactivated the BZLF1 promoter but not the BRLF1 promoter (Fig. 7a). Conversely, XBP-1s in combination with PKD weakly transactivated the BRLF1 promoter (Fig. 7a). In order to ensure that the lack of a robust effect from PKD is not cell type specific, we performed the luciferase assay in HeLa cells for the Zp. Here, XBP-1s and active PKD alone do not transactivate Zp, but together weakly transactivate Zp (Fig. 7b). However, these effects are not statistically significant (*P*>0.05, two-tailed *t*-test). At all times BZLF1 and BRLF1 were able to transactivate their respective promoters.

Importantly, overexpressing either PKD or XBP-1s alone or together in JSC-1 cells did not induce BZLF1 or BRLF1 protein expression (Fig. 7c), whereas K-RTA expression was induced whenever XBP-1s was overexpressed. Q-RT-PCR confirmed that the mRNA expression of BZLF1 and BRLF1 did not increase when PKD was expressed with and without XBP-1s (Fig. 7d). K-RTA mRNA expression increased significantly (*P*<0.05, two-tailed *t*-test) whenever XBP-1s was overexpressed (Fig. 7d). Therefore, active PKD alone does not lead to EBV BZLF1 and EBV BRLF1, or KSHV K-RTA expression. Also, active PKD together with XBP-1s does not induce EBV BZLF1 and BRLF1 expression.

## DISCUSSION

Plasma cell terminal differentiation and the associated expression of XBP-1s are linked to EBV and KSHV lytic cycle induction (Bhende *et al.*, 2007; Laichalk & Thorley-Lawson, 2005; Sun & Thorley-Lawson, 2007; Wilson *et al.*, 2007; Yu *et al.*, 2007). Recently this has been extended beyond human gammaherpesviruses with the demonstration that plasma cells account for most of the lytic reactivation of murine herpesvirus 68 in mice (Liang *et al.*, 2009). In EBV and KSHV co-infected PEL however, selective induction of one viral lytic cycle cross-represses the other viral lytic cycle and co-expression of both KSHV and EBV immediate–early proteins results in mutual inhibition (Jiang *et al.*, 2008; Miller *et al.*, 1997; Xu *et al.*, 2007). This suggests that a selective lytic switch that induces one or other virus is required for successful EBV or KSHV lytic replication in PEL. Here we show that XBP-1s is able to induce KSHV lytic replication alone in dually infected PEL. XBP-1s does not induce the expression of EBV BZLF1 and BRFL1 immediate–early–proteins or transcripts in PEL or BL. XBP-1s with PKD also does not induce the expression of BZLF1 and BRFL1 immediate–early proteins or transcripts in PEL.

Previous studies have suggested XBP1s, either alone or in combination with PKD, can activate the EBV lytic cycle (Bhende *et al.*, 2007; Sun & Thorley-Lawson, 2007); however, the mechanistic detail is unclear. Sun & Thorley-Lawson (2007) showed that, in HeLa cells, XBP-1s cannot transactivate Zp. Whereas, Bhende *et al.* (2007) showed, also in HeLa cells, that XBP-1s weakly transactivates Zp and Rp, and the effect on Zp could be enhanced by co-expression of constitutively active PKD (Bhende *et al.*, 2007; Sun & Thorley-Lawson, 2007). Our data in HEK 293T cells supports the observations that XBP-1s alone can weakly transactivate Zp and Rp. Similarly, we show co-expression of XBP-1s and PKD weakly transactivates Zp in HEK 293T and HeLa cells, although not to the magnitude described previously. This may reflect differences in assay sensitivity or may reflect the fact that we have used human XBP-1s in our studies rather than the murine XBP-1s used by Bhende *et al.* (2007). Nevertheless, the relevance of these observations in HEK 293T and HeLa cells, and the response of EBV to XBP-1s and PKD in B-cell tumour lines, is questionable.

The ability of XBP-1s to transactivate either Zp or Rp probably depends on both the cell type and on the nature of the individual cell lines. This is supported by observations that distinct chemical inducing agents have different effects on the induction of EBV and KSHV lytic cycles in various lymphoma lines (Countryman *et al.*, 2008; Miller *et al.*, 1997). In a lymphoblastoid and a nasopharyngeal carcinoma cell line XBP-1s and PKD clearly activated EBV BZLF1 protein expression, but BL or PEL cell lines were not tested (Bhende *et al.*, 2007). In an unusual multiple-myeloma cell line latently infected with EBV, only low levels of BZLF1 transcript could be induced by XBP-1s. This observation contrasts with recent data indicating that EBV can latently infect multiple myeloma cell lines *in vitro.* In these circumstances the endogenous, active XBP-1s does not drive the EBV lytic cycle (Anastasiadou *et al.*, 2009). Here we show that despite transactivation of Zp and Rp by XBP-1s in HEK 293T cells, there are no detectable levels of BZLF1 and BRLF1 mRNA or proteins after XBP-1s overexpression in B-cell tumour lines.

Why EBV Zp is refractory to XBP-1s in PEL and BL, even when PKD is co-expressed, is not clear, although the role of the chromatin structure at Zp is likely to be important. Indeed, the binding of myocyte enhancer factor 2D to Zp recruits class II histone deacetylases (HDACs), which presumably promote a repressed chromatin structure on Zp in lymphoma cell lines (Gruffat *et al.*, 2002; McDonald *et al.*, 2010). Derepression of Zp-associated chromatin by PKD was suggested as the mechanism that allows XBP-1s to activate Zp (Bhende *et al.*, 2007). PKD is a member of the serine/threonine protein kinase family (Rey *et al.*, 2006) which can be activated by protein kinase C (PKC) (Zugaza *et al.*, 1996), a cellular target of phorbol esters such as TPA (Wang, 2006). PKD can also be activated by BCR cross-linking via the PKC pathway (Matthews *et al.*, 2000). In our hands, TPA induces both K-RTA and BZLF1 in PEL and BCR cross-linking induces BZLF1 in BL. Activated PKD can phosphorylate and therefore inactivate class IIa HDACs; this is similar to the effect of HDAC inhibitors (HDACi), such as trichostatin A (Chang & Liu, 2000), VPA (Feng & Kenney, 2006) and sodium butyrate (NaB). As HDACi are also capable of inducing EBV lytic reactivation in various cell lines (Gradoville *et al.*, 2002), we investigated whether XBP-1s is also induced by any of these chemical inducers. Here we show that neither NaB, VPA nor TPA induce activated XBP-1s in PEL, and therefore the effects of HDACi and TPA on the Zp is independent of XBP-1s.

BCR cross-linking induces the EBV lytic cycle and can also transiently induce XBP-1s production. However, this transient XBP-1s cannot be unambiguously linked to BZLF1 expression (McDonald *et al.*, 2010). In this study we used DTT to chemically induce XBP-1s in PEL and BL cell lines. Although DTT is able to induce XBP-1 splicing and K-RTA in an XBP-1s dependent manner in HEK 293T cells (Wilson *et al.*, 2007), it causes a large amount of cell death in PEL cells, making it impossible to determine K-RTA, BZLF1 or BRFL1 protein expression. Nevertheless, K-RTA mRNA expression was detected following DTT treatment of PEL (Fig. 6b). Also DTT does not cause cell death in BL cells; it induces XBP-1s production but does not induce BZLF1 expression. Taken together these data suggest that induction of the EBV lytic cycle in lymphoma cell lines of PEL and BL is not linked to XBP-1s activity, although the effects in B-cells *in vivo* may be different.

## METHODS

### Cell culture.

The PEL cell line JSC-1 and the BL cell lines Mutu, Daudi and Akata were grown in RPMI 1640 medium (Invitrogen) with 10 % FCS (BioSera), 100 units penicillin ml^−1^ and 100 units streptomycin ml^−1^ (Invitrogen) at 37 °C in 5 % CO_2_. All superinfected PEL cell lines, CRO6 clone 2, BC3 clone 6 and BC3 clone 10 (a kind gift from Pankaj Treviti), were grown with G418 selection as described previously (Xu *et al.*, 2007). HEK 293T cells and HeLa cells were grown in Dulbecco's modified Eagle's medium (Invitrogen) with 15 and 10 % FCS, respectively, 100 units penicillin ml^−1^ and 100 units streptomycin ml^−1^ (Invitrogen) at 37 °C in 10 % CO_2_. To induce BZLF1 and K-RTA expression, cells were cultured with the following concentrations of inducing agents: DTT, 2 mM; goat anti-human IgG antibody (Sigma), 5 μg ml^−1^; 12-*O*-tetradecanoyl-phorbol 13-acetate (TPA; Sigma), 20 ng μl^−1^; VPA (Merck), 1.5 mM.

### Lentiviral vector construction and transduction.

The lentiviral vector construction of pXBP1sIG and pIG has been described previously (Wilson *et al.*, 2007). The lentiviral vector for activated PKD, pPKDm-IG, was produced from the pHA.PKD.S738A/S742A plasmid kindly supplied by Alex Toker (Storz & Toker, 2003) (Addgene). Briefly, *Bam*HI and *Xho*I were used to clone the HA.PKD.S738A/S742A insert into the lentiviral vector pIG to produce the pPKDm–IG construct. To produce lentiviral viruses, HEK 293T cells were transfected with 1 μg pMDG, 1 μg p8.91 and 1.5 μg of lentiviral vector, by using FuGENE-6 (Roche). The supernatants were collected 48 and 72 h post-transfection and filtered. Both PEL and BL cell lines (5×10^4^ cells per well in a 24-well plate) were transduced with lentivirus vector at an input equivalent to an m.o.i. of 2 or 5 as measured in JSC-1 cells. This resulted in an actual level of infection of 60–80 % in PEL and 10–25 % in BL cell lines. Lentiviruses were added to the cells, spinoculated for 1 h, at 500 ***g*** and room temperature. No selection for infected cells was used after the transduction. Forty-eight hours after transduction the cells were analysed using flow cytometry.

### RNA extraction and reverse-transcriptase PCR (RT-PCR).

Total RNA was purified from 8–10×10^5^ cells resuspended in TRIzol (Invitrogen). The TRIzol mixture was first treated with chloroform and RNA was isolated using an RNA extraction kit (Qiagen), including an on-column DNase (Promega) digestion. Reverse transcription was carried out using Ominiscript Reverse Transcriptase (Qiagen) according to the manufacturer's instructions with 1–2 μg total RNA.

### PCR and restriction digestion.

Oligo-dT (Promega)-primed cDNA was used for PCR amplification across the XBP-1 intron as described previously by Wilson *et al.* (2007). The PCR product was then digested with *Pst*I for 1 h at 37 °C.

### Q-RT-PCR for mRNA.

Q-RT-PCR was performed with a QuantiTect SYBR Green PCR kit (Qiagen) using the following primers: BZLF1 (5′-CTATCAGGACCTGGGAGGGC-3′ and 5′-CACAGCACACAAGGCAAAGG-3′) (Schelcher *et al.*, 2005), BRLF1 (5′-AATTTACAGCCGGGAGTGTG-3′ and 5′-AGCCCGTCTTCTTACCCTGT-3′) (Chia *et al.*, 2008), K-RTA (5′-TTGGTGCGCTATGTGGTCTG-3′ and 5′-GGAAGGTAGACCGGTTGGAA-3′) (Caselli *et al.*, 2005) and L32 (5′-CAACATTGGTTATGCAAGCAACA-3′ and 5′-TGACGTTGTGGACCAGGAACT-3′) (Schelcher *et al.*, 2005). L32 is a cellular ribosomal gene used to assess the preparation of cDNA and all the mRNA levels were normalized with L32. The PCR was prepared according to the manufacturer's instructions and was performed by using an ABI Prism 7000 (Applied Biosystems) with the following programme: 50 °C for 2 min, 95 °C for 15 min, 95 °C for 15 s and 60 °C for 1 min; the third and fourth steps are then repeated for 40 cycles.

### Luciferase promoter assays.

HEK 293T cells or HeLa cells were plated at a density of 2×10^4^ cells per well in 96-well plates. Cells were transfected the next day with 20 ng of the appropriate reporter and expression constructs by using fuGENE-6 (Roche). The ORF50 reporter plasmids were a kind gift from Erle Robertson (Robertson & Ambinder, 1997). The BZLF1 promoter and expression vectors have been described previously (Bryant & Farrell, 2002) and the BRLF1 promoter and expression constructs were a kind gift from Shannon Kenney (Bhende *et al.*, 2007). The pXBP1sIG and pIG plasmids were described previously (Wilson *et al.*, 2007). Co-transfection of 2 ng of Renilla expression vector (a kind gift from Professor Gary Stein, University of Massachusetts, MA, USA) was also used for monitoring transfection efficiency. After 48 h, the relative light units (RLU) and the expression of the Renilla construct were determined using luciferase Stop & Glo reagents (dual-luciferase assay kit; Promega) according to the manufacturer's instructions. Data were read using a GloMaz96 microplate luminometer (Promega) with a single injector. All assays were performed in triplicate.

### Methylation of promoter luciferase constructs.

BRLF1 promoter was methylated using CpG methyltransferase (New England Biolabs) according to the manufacturer's instructions. Methylation of the plasmids was confirmed by performing restriction enzyme digestion with methylation-sensitive enzymes. Rp was digested with *BgI*I and *Mln*I.

### Immunoblotting.

Forty-eight hours after transduction (with no selection), the samples were lysed with a mixture of 1 M DTT and sample buffer (1 : 3 ratio) (0.2 M Tris-HCL pH 6.8, 5.2 % SDS, 20 % Glycerol and bromophenol blue) then sonicated and heated to 95 °C for 5 min. Proteins were resolved on 10 % polyacrylamide gels by SDS-PAGE and transferred to a PVDF membrane. The membrane was blocked with 5 % non-fat dried milk powder (NFDM) in TBS solution containing 0.1 % Tween 20 (Sigma) (TBS-T) for 1 h. The membrane was then probed with primary antibody as follows: BZLF1, BZ-1 diluted 1 : 2000 (Bryant & Farrell, 2002); tubulin, anti-*α*-tubulin clone DM1A (Sigma) diluted 1 : 40,000; K-RTA polyclonal, kind gift of Don Ganem (Lukac *et al.*, 1998), diluted 1 : 40 000; PKD, PKC μ(D-20), sc-935 (Santa Cruz) diluted 1 : 2000; BRLF1, anti-EBV transcription factor R (Argene) diluted 1 : 2000; BMRF1, EBV early antigen diffuse (Vector Laboratories) diluted 1 : 2000; XBP-1s, a kind gift from Giovanna Roncador, diluted 1 : 50 (Maestre *et al.*, 2009), all in 1 % NFDM in TBS-T solution at 4 °C overnight. The membrane was then incubated with appropriate HRP-conjugated secondary antibodies (GE Healthcare) in 1 % NFDM TBS-T solution for 1 h at room temperature. The blots were washed five times for 5 min periods in TBS-T before developing, and were visualized using ECL Western blotting detection reagents or ECL Advanced Western blotting detection reagents (GE Healthcare).
